# The burden of the knowledge-to-action gap in acute appendicitis

**DOI:** 10.1007/s00464-023-10449-4

**Published:** 2023-10-26

**Authors:** Stefano Piero Bernardo Cioffi, Michele Altomare, Mauro Podda, Andrea Spota, Stefano Granieri, Elisa Reitano, Beatrice Zamburlini, Francesco Virdis, Roberto Bini, Shailvi Gupta, Guido Torzilli, Andrea Mingoli, Osvaldo Chiara, Stefania Cimbanassi, The REsiDENT-1 research group, Giulia Arianna Abruzzese, Francesca Albanesi, Erika Andreatta, Ludovica Baldari, Laura Benuzzi, Emanuele Bevilaqua, Alessandro Michele Bonomi, Greta Brachetti, Giulia Cannavale, Andrea Piero Chierici, Riccardo Cirelli, Gaia Colletti, Vera D’abrosca, Piergiorgio Danelli, Luca Del Prete, Francesco Di Capua, Francesca Di Vittorio, Davide Ferrari, Luca Ferrario, Laura Fiore, Colomba Frattaruolo, Caterina Froiio, Ludovica Gibelli, Irene Giusti, Ugo Giustizieri, Samuele Grandi, Giulio Iacob, Alessia Kersik, Pietro Lombardi, Marco Longhi, Leonardo Lorusso, Michele Manara, Elena Manzo, Jacopo Nicolo Marin, Marianna Maspero, Valentina Messina, Pamela Milito, Mattia Molteni, Eleonora Monti, Vincenzo Nicastro, Giorgio Novelli, Sissi Paleino, Silvia Pavesi, Carolina Perali, Isabella Pezzoli, Roberta Ragozzino, Giuliano Santolamazza, Luca Scaravilli, Gilda Tornatore, Francesco Toti, Vincenzo Tripodi, Elisa Vaterlini, Barbara Vignati, Cecilia Maina, Alessandra Borghi, Marco Realis Luc, Paolo Pizzini, Riccardo Masserano, Marta Maistri, Laura Traballi, Francesco Cammarata, Alvino Boero, Davide Socci, Margherita Carbonaro, Martina Pellegrini

**Affiliations:** 1 Advanced Technologies in Surgery, Department of Surgical Sciences, University of Rome Sapienza, Rome, Italy; 2 General Surgery Trauma Team, ASST GOM Niguarda, Viale Ettore Majorana, 20162 Milan, Italy; 3 Department of Surgical Sciences, Cagliari State University, Cagliari, Italy; 4 General Surgery Unit, ASST-Brianza, Vimercate Hospital, Vimercate, Italy; 5 Division of General Surgery, Department of Translational Medicine, Maggiore Della Carità Hospital, University of Eastern Piedmont, Novara, Italy; 6 Research Institute Against Digestive Cancer, IRCAD, Strasbourg, France; 7 University of Maryland, Baltimore, USA; 8 Humanitas University, Rozzano, Italy; 9 Department of Pathophysiology and Transplantation, University of Milan, Milan, Italy; 10 General Surgery Residency Program, University of Milan, Milan, Italy

**Keywords:** Acute appendicitis, Compliance, Evidence-based surgery, Guidelines, Knowledge-to-action gap, Knowledge transfer

## Abstract

**Background:**

The burden of emergency general surgery (EGS) is higher compared to elective surgery. Acute appendicitis (AA) is one of the most frequent diseases and its management is dictated by published international clinical practice guidelines (CPG). Adherence to CPG has been reported as heterogeneous. Barriers to clinical implementation were not studied. This study explored barriers to adherence to CPG and the clinico-economic impact of poor compliance.

**Methods:**

Data were extracted from the three-year data lock of the REsiDENT-1 registry, a prospective resident-led multicenter trial. We identified 7 items from CPG published from the European Association of Endoscopic Surgery (EAES) and the World Society of Emergency Surgery (WSES). We applied our classification proposal and used a five-point Likert scale (Ls) to assess laparoscopic appendectomy (LA) difficulty. Descriptive analyses were performed to explore compliance and group comparisons to assess the impact on outcomes and related costs. We ran logistic regressions to identify barriers and facilitators to implementation of CPG.

**Results:**

From 2019 to 2022, 653 LA were included from 24 centers. 69 residents performed and coordinated data collection. We identified low compliance with recommendations on peritoneal irrigation (PI) (25.73%), abdominal drains (AD) (34.68%), and antibiotic stewardship (34.17%).

Poor compliance on PI and AD was associated to higher infectious complications in uncomplicated AA. Hospitalizations were significantly longer in non-compliance except for PI in uncomplicated AA, and costs significantly higher, exception made for antibiotic stewardship in complicated AA. The strongest barriers to CPG implementation were complicated AA and technically challenging LA for PI and AD. Longer operative times and the use of PI negatively affected antibiotic stewardship in uncomplicated AA. Compliance was higher in teaching hospitals and in emergency surgery units.

**Conclusions:**

We confirmed low compliance with standardized items influenced by environmental factors and non-evidence-based practices in complex LA. Antibiotic stewardship is sub-optimal. Not following CPG may not influence clinical complications but has an impact in terms of logistics, costs and on the non-measurable magnitude of antibiotic resistance. Structured educational interventions and institutional bundles are required**.**

**Supplementary Information:**

The online version contains supplementary material available at 10.1007/s00464-023-10449-4.

Emergency General Surgery (EGS) is a public health issue with a higher burden compared to elective surgery. EGS patients account for 11% of surgical admissions per year and 50% of the overall surgical mortality. EGS admissions are 3 times higher than strokes and congestive heart failure and 2 times higher than new cancer diagnosis per year [1–5].

Acute appendicitis (AA) is one of the most contributing factors to the magnitude of EGS [6]. Current clinical practice guidelines (CPG) developed by the World Society of Emergency Surgery (WSES) in 2020 and the European Association of Endoscopic Surgery (EAES) in 2016 provide recommendations for AA diagnosis and management [7, 8].

The real-world adherence with recently published CPG for the management of AA in adult patients has been explored recently in a multinational snapshot by the European Society for Surgery of Trauma (ESTES), which shows better compliance with selected outcomes and highlights-specific knowledge-to-action (KTA) gaps related to worse outcomes [9].

Compliance with recommendations on peritoneal irrigation (PI) and abdominal drainage (AD) has not been studied. Moreover, the authors did not explore any barrier limiting compliance with recommendations; thus, no clinical bundles to be implemented were proposed.

The KTA gap has also been studied in paediatric appendicitis and acute biliary diseases, reporting low compliance potentially related to worst outcomes [10–12].

Our working hypothesis was that the KTA in acute appendicitis is influenced by environmental, patient-related and surgeon-related factors and is a contributor to the burden of EGS.

This study aimed to identify possible barriers to the application of evidence-based surgery (EBS) principles by evaluating adherence to the most cited CPG on AA exploring the impact of low compliance.

## Methods

### Primary endpoint

- Identify predictive factors for compliance with selected items of CPG [7, 8].

### Secondary endpoints

- Assess the KTA in AA exploring compliance to selected items.
- Quantify the clinical and economic impact of poor compliance.

### Study design and setting

Data were reported according to the STROBE statement for observational studies [13].

Data were extracted from a three-year data lock of REsiDENT-1 trial registry to perform a spin-off analysis. This is a multicenter project, started in October 2019, approved by the ethics committee of the ASST GOM Niguarda Coordinating Center. Local registration number n° 486-22072021, ClinicalTrials.gov: NCT05075252. All the centers re-evaluated the protocol before the inclusion.

The REsiDENT-1 project aims to standardize the reporting of AA severity and the grade of peritoneal contamination and explore the relationships between PI and postoperative intraabdominal abscesses [14].

Residents belong to the General Surgery Residency program of the University of Milan and do a clinical rotation every 6 months to 1 year. The online guidance regarding the compilation of the database was performed with group webinars led by seniors. A regular checkpoints and data cleaning is performed every 6 months by the steering committee.

### Patient enrolment

*The inclusion criteria* were age > 18 years, laparoscopic appendectomy (LA), and a histological diagnosis of AA.

*Exclusion criteria* conversion to open surgery and other primary causes of intra-abdominal infection clinically mimicking acute appendicitis (i.e. right colonic diverticulitis, gynaecological diseases).

### Variables of interest

We identified seven items from the published CPG (Table 1).

**Table 1:** Summary of the selected Items from published guidelines on the management of acute appendicitis

	Compliance to guidelines in Acute Appendicitis		
	Item WSES 2020		Item EAES 2015
	Preoperative		
1.9	We suggest that cross-sectional imaging (i.e., CT scan) in high-risk patients younger than 40 years old (with AIR score 9–12 and Alvarado score 9–10 and AAS ≥ 16) may be avoided before proceeding to diagnostic + / − therapeutic laparoscopy [QoE: Moderate; Strength of recommendation: Weak; 2B]		
1.10	We recommend POCUS as the most appropriate first-line diagnostic tool in both adults and children, if an imaging investigation is indicated based on clinical assessment [QoE: Moderate; Strength of recommendation: Strong; 1B]	1	Ultrasound is reliable in increasing the likelihood of acute appendicitis, but is not reliable to exclude the diagnosis
3.2	We recommend against delaying appendectomy for acute appendicitis needing surgery beyond 24 h from the admission [QoE: Moderate; Strength of recommendation: Strong; 1B]		
	Intraoperative		
4.8	We recommend performing suction alone in complicated appendicitis patients with intra-abdominal collections undergoing laparoscopic appendectomy [QoE: Moderate; Strength of recommendation: Strong; 1B]	12	In general, meticulous suction of intra-peritoneal fluid or collection is suggested, the philosophy should be: " leave no pus behind". Routine use of drains in appendectomy is not recommended SOR Weak/Strong
4.12	We recommend against the use of drains following appendectomy for complicated appendicitis in adult patients [QoE: Moderate; Strength of recommendation: Strong; 1B]		
	Postoperative		
7.1	We recommend a single preoperative dose of broad spectrum antibiotics in patients with acute appendicitis undergoing appendectomy. We recommend against postoperative antibiotics for patients with uncomplicated appendicitis [QoE: High; Strength of recommendation: Strong; 1A]	2	No evidence of routine postoperative antibiotics in uncomplicated appendicitis SOR Strong
7.2	We recommend against prolonging antibiotics longer than 3–5 days postoperatively in case of complicated appendicitis with adequate source-control [QoE: High; Strength of recommendation: Strong; 1A]	6	In complicated appendicitis postoperative antibiotics are recommended SOR Strong

*EAES* European Association of Endoscopic Surgery, *POCUS* point of care ultrasound, *AIR* appendicitis inflammatory response, *AAS* adult appendicitis score, *QOE* quality of evidence, *WSES* World Society of Emergency Surgery

An evidence-based surgery pathway was identified in patients with uncomplicated AA considering the combined adherence to recommendation on timing of surgery, peritoneal irrigation, abdominal drainage and antimicrobial therapy (AMT) stewardship. The basic assumption is that patients with uncomplicated appendicitis should benefit from shorter procedures and hospitalizations.

Variables of interest in the registry included clinical, intraoperative, and postoperative data [14].

Acute appendicitis severity was reported following our published intraoperative classification [14]. Its clinical efficacy in identifying complex and simple diseases, using histology as the gold standard, is good to moderate and has been discussed in a previous published study [15]. The classification is reported below.

### Appendix aspect

- Erythematous and oedematous appendix
- Appendiceal phlegmon
- Gangrenous appendix
- Perforated appendix

### Contamination

- Single abscess
- Multiple abscess
- Localized purulent peritonitis
- Diffuse purulent peritonitis
- Localized faecal peritonitis
- Diffuse faecal peritonitis

The technical difficulty of LA was defined following a 5-point Likert-type scale [16], considering the progressive operative autonomy of the operator from a procedure performed independently (1), via procedures requiring passive (2) and active (3) assistance from the assistant, to complex, challenging procedures needing an external, non-scrubbed (4) or scrubbed (5), help to finish the LA.

We explored three different areas of possible barriers to the implementation of the CPG in daily surgical practice:

- Environmental: hospital academic status, dedicated EGS service, timing of surgery
- Patient-related: clinical data, appendicitis severity, peritoneal contamination, operative time
- Surgeon-related: technical difficulty, operative time

Health-care related expenditures per each hospitalization were calculated following the costs related to each of the Diagnosis Related Group of interest for appendectomy, considering complicated and uncomplicated forms, with or without postoperative complications and the duration of the hospitalization [17].

### Statistical analysis

Numeric variables are expressed as mean (± SD) and discrete outcomes as absolute and relative (%) frequencies. Student’s *t* test, Welch’s *t* test, or Mann–Whitney *U* test according to data distribution were applied for comparisons of continuous variables. Discrete outcomes were compared using the Chi-square or Fisher’s exact test.

We described compliance with the selected items from the CPG.

Univariate analyses were performed to identify any differences in terms of clinical outcomes and health-care-related costs considering compliance to different items. Patients were stratified according to the severity of the appendicitis, complicated AA (perforated AA, intra-abdominal abscess, peritonitis) and uncomplicated AA (erythematous, phlegmonous and gangrenous).

The CPG items 4.8–12, 4.12–12, and 7.1–2 were explored using a multivariate logistic regression model to assess their relationship with environmental and clinical variables. Data were checked for multicollinearity using the Belsley–Kuh–Welsch technique. Heteroskedasticity and normality of residuals were assessed using the White test and Shapiro–Wilk test, respectively. The confidence interval (CI) was set at 95%. The alpha risk was set at 5% and two-tailed tests were used. Statistical analyses were performed using EasyMedStat (version 3.20.4; www.easymedstat.com).

## Results

### Population analysis

**Fig. 1:**
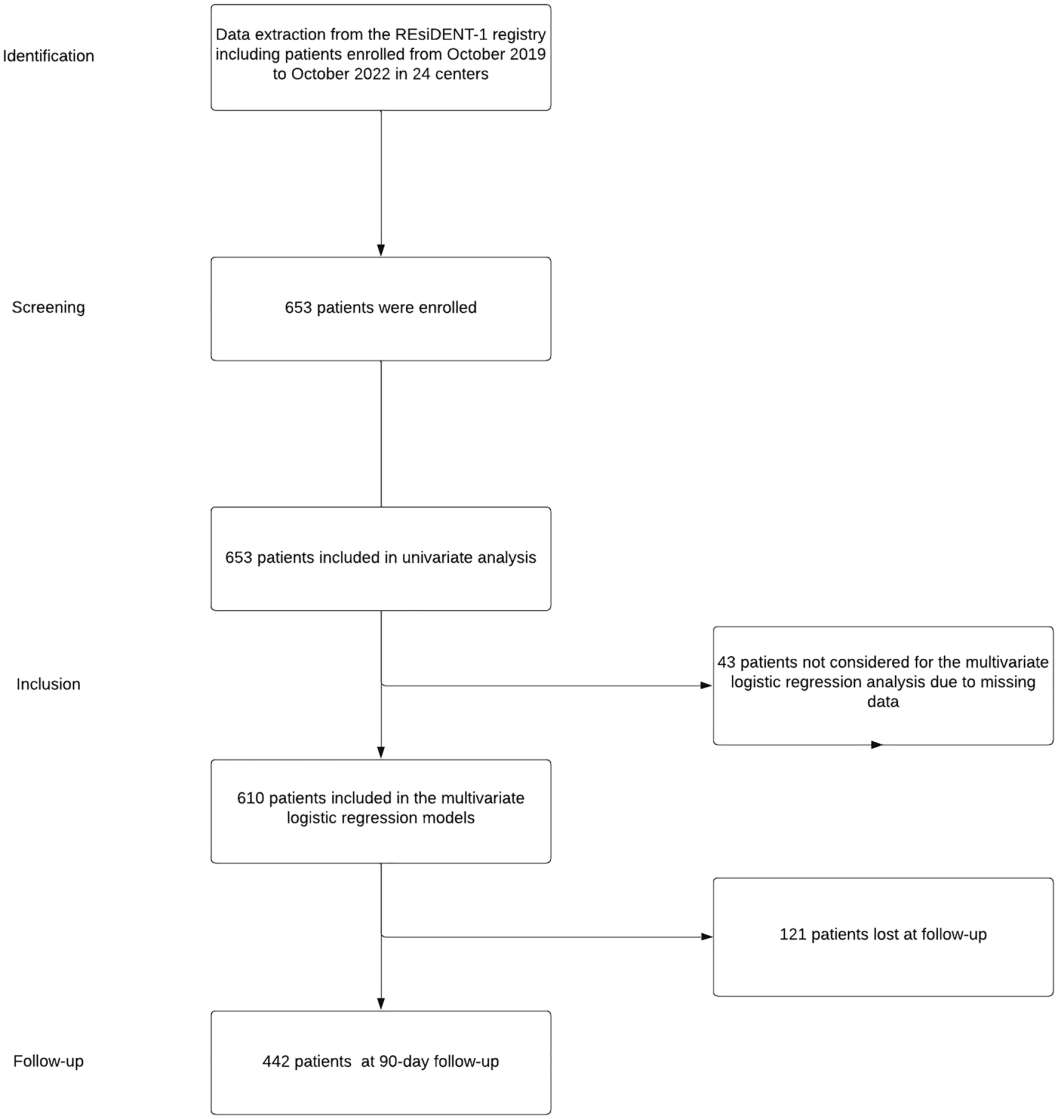
Flowchart of patients’ enrolment following the STROBE guidelines

A total of six-hundred fifty-three patients enrolled from October 2019 to October 2022 who met the inclusion criteria were considered for this analysis. Sixty-nine general surgery residents from 24 hospitals participated in the study (See Supplementary Materials for the full list). The 90 days follow up rate was 78.6%. The flowchart of patient enrolment is reported in Fig. 1.

### Compliance assessment, clinical and economic outcomes

**Fig. 2:**
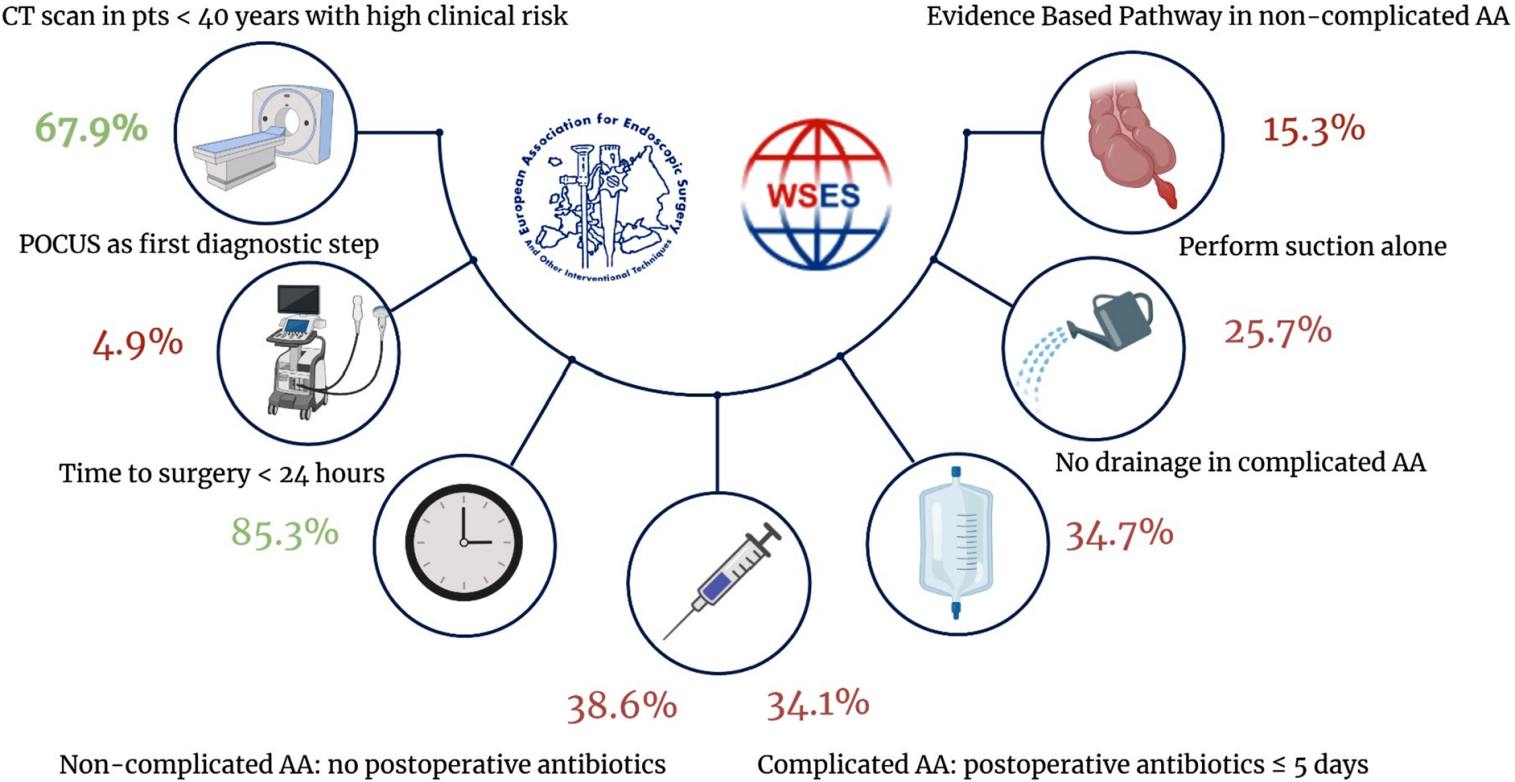
Representation of the compliance assessment to the clinical practice guidelines published by the World Society of Emergency Surgery and the European Society for Endoscopic Surgery. Each explored item from the guidelines is reported with the respective compliance. The Evidence Based Pathway in uncomplicated AA results from the integration of compliance with items on: timing of surgery, peritoneal irrigation, abdominal drainage and antibiotic stewardship. This pathway was analysed since uncomplicated AA can be a mild disease, amenable of operative and non-operative treatment in which hospitalizations, costs and morbidity should be kept as lower as possible.

The results of the adherence analysis are reported in Fig. 2.

Focusing on clinical and health-care-related outcomes, poor compliance to guidelines was associated with significantly higher rates of Surgical Site Infections (SSI), specifically Organ Space (OS) infections, with the inappropriate use of peritoneal irrigation and abdominal drainage in uncomplicated appendicitis, 6 OS (5.04%) versus 1 OS (0.62%), *p* = 0.04 for both groups. Per each of the items considered, poor compliance was associated with significantly longer hospitalizations, except in case of non-compliance with drain placement in uncomplicated AA.

Low adherence to CPG impacted health-related expenditures with significant higher costs in case of noncompliance for all items, exception made for non-adherence to CPG on antibiotic stewardship in complicated AA.

The whole comparison between groups of patients in which single items of the guidelines were followed or not, stratified considering AA severity, is reported in Table 2.

**Table 2:** Group comparisons, stratifying patients for appendicitis severity, considering compliance to selected CPG items

Variable	Avoidance of peritoneal irrigation						Avoidance of abdominal drainage					
	Complicated AA			Non-complicated AA			Complicated AA			Non-complicated AA		
	Yes *N* = 48	No *N* = 324	*p*-Value	Yes *N* = 119	No *N* = 161	*p*-Value	Yes *N* = 129	No *N* = 243	*p*-Value	Yes *N* = 175	No *N* = 105	*p*-Value
Academic hospital			0.10			** < 0.001**			** < 0.001**			** < 0.001**
Yes	23 (47.92%)	112 (34.57%)		57 (47.9%)	43 (26.71%)		64 (49.61%)	71 (29.22%)		83 (47.43%)	17 (16.19%)	
No	25 (52.08%)	212 (65.43%)		62 (52.1%)	118 (73.29%)		65 (50.39%)	172 (70.78%)		92 (52.57%)	88 (83.81%)	
Dedicated EGS service			**0.03**			** < 0.001**			** < 0.001**			** < 0.001**
Yes	21 (43.75%)	89 (27.47%)		55 (46.22%)	30 (18.63%)		59 (45.74%)	51 (20.99%)		71 (40.57%)	14 (13.33%)	
No	27 (56.25%)	235 (72.53%)		64 (53.78%)	131 (81.37%)		70 (54.26%)	192 (79.01%)		104 (59.43%)	91 (86.67%)	
Age	34.6 (± 13.53)	41.74 (± 16.94)	**0.01**	34.42 (± 15.01)	33.12 (± 15.07)	0.33	35.83 (± 15.18)	43.47 (± 16.89)	** < 0.001**	33.7 (± 13.69)	33.64 (± 17.1)	0.17
BMI	23.98 (± 3.96)	24.65 (± 4.16)	0.47	24.04 (± 3.61)	23.91 (± 3.45)	0.92	23.33 (± 3.78)	25.2 (± 4.18)	** < 0.001**	23.64 (± 3.44)	24.52 (± 3.58)	**0.03**
ASA			0.12			0.98			** < 0.001**			0.68
1	30 (62.5%)	160 (49.54%)		79 (66.39%)	108 (67.08%)		86 (66.67%)	104 (42.98%)		120 (68.57%)	67 (63.81%)	
2	18 (37.5%)	138 (42.72%)		35 (29.41%)	47 (29.19%)		38 (29.46%)	118 (48.76%)		49 (28.0%)	33 (31.43%)	
3	0 (0.0%)	23 (7.12%)		5 (4.2%)	6 (3.73%)		5 (3.88%)	18 (7.44%)		6 (3.43%)	5 (4.76%)	
4	0 (0.0%)	2 (0.62%)					0 (0.0%)	2 (0.83%)				
Charlson comorbidity index	0.167 (± 0.519)	0.715 (± 1.25)	**0.00**	0.345 (± 0.961)	0.398 (± 1.15)	0.68	0.364 (± 1.09)	0.793 (± 1.22)	** < 0.001**	0.297 (± 0.811)	0.505 (± 1.4)	0.37
Alvarado score	6.33 (± 1.86)	6.92 (± 1.53)	**0.04**	5.94 (± 1.8)	6.06 (± 1.6)	0.84	6.52 (± 1.59)	7.02 (± 1.56)	**0.001**	6.0 (± 1.8)	6.03 (± 1.49)	0.97
AIR score	5.63 (± 2.08)	6.58 (± 1.93)	**0.01**	4.9 (± 1.83)	5.35 (± 1.71)	**0.03**	5.83 (± 2.04)	6.79 (± 1.86)	** < 0.001**	5.14 (± 1.78)	5.17 (± 1.77)	> 0.999
Operative time (minutes)	66.28 (± 22.73_)_	76.47 (± 29.5)	0.05	60.87 (± 24.01)	59.84 (± 19.06)	0.91	67.13 (± 22.81)	79.41 (± 30.86)	** < 0.001**	58.65 (± 19.51)	62.98 (± 23.77)	0.28
Difficulty grade			** < 0.001**			0.19			** < 0.001**			** < 0.001**
1	18 (37.5%)	30 (9.26%)		48 (40.34%)	45 (27.95%)		35 (27.13%)	13 (5.35%)		76 (43.43%)	17 (16.19%)	
2	14 (29.17%)	92 (28.4%)		47 (39.5%)	83 (51.55%)		46 (35.66%)	60 (24.69%)		76 (43.43%)	54 (51.43%)	
3	11 (22.92%)	105 (32.41%)		21 (17.65%)	28 (17.39%)		36 (27.91%)	80 (32.92%)		22 (12.57%)	27 (25.71%)	
4	1 (2.08%)	80 (24.69%)		2 (1.68%)	4 (2.48%)		5 (3.88%)	76 (31.28%)		0 (0.0%)	6 (5.71%)	
5	1 (2.08%)	15 (4.63%)		1 (0.84%)	1 (0.62%)		3 (2.33%)	13 (5.35%)		1 (0.57%)	1 (0.95%)	
Length of stay (days)	3.02 (± 1.02)	4.96 (± 2.33)	** < 0.001**	3.37 (± 1.52)	3.59 (± 1.35)	0.07	3.84 (± 1.92)	5.18 (± 2.36)	** < 0.001**	3.18 (± 1.19)	4.03 (± 1.62)	** < 0.001**
Clavien dindo classification of surgical complications > 2			0.60			> 0.999			0.27			0.38
Yes	0 (0.0%)	8 (2.47%)		1 (0.62%)	0 (0.0%)		1 (0.78%)	7 (2.88%)		0 (0.0%)	1 (0.95%)	
No	48 (100.0%)	316 (97.53%)		160 (99.38%)	119 (100.0%)		128 (99.22%)	236 (97.12%)		175 (100.0%)	104 (99.05%)	
Comprehensive complications index	3.74 (± 6.37)	6.31 (± 11.74)	0.27	3.12 (± 5.12)	3.76 (± 7.09)	0.86	4.51 (± 7.75)	6.76 (± 12.62)	0.18	3.36 (± 5.59)	3.69 (± 7.42)	0.52
Health-care costs	3558.83 (± 859.76)	3911.44 (± 888.28)	**0.02**	2556.44 (± 363.98)	2668.94 (± 379.83)	**0.02**	3702.5 (± 881.76)	3952.72 (± 886.09)	**0.01**	2586.6 (± 354.25)	2678.68 (± 406.56)	**0.04**
Unplanned readmission			0.61			0.09			0.28			0.09
Yes	0 (0.0%)	9 (3.69%)		5 (5.05%)	1 (0.75%)		1 (1.14%)	8 (4.19%)		1 (0.75%)	5 (5.05%)	
No	35 (100.0%)	235 (96.31%)		94 (94.95%)	133 (99.25%)		87 (98.86%)	183 (95.81%)		133 (99.25%)	94 (94.95%)	
SSI overall			0.80			**0.04**			0.72			**0.04**
Yes	4 (8.33%)	35 (10.8%)		1 (0.62%)	6 (5.04%)		12 (9.3%)	27 (11.11%)		1 (0.62%)	6 (5.04%)	
No	44 (91.67%)	289 (89.2%)		160 (99.38%)	113 (94.96%)		117 (90.7%)	216 (88.89%)		160 (99.38%)	113 (94.96%)	
OS overall			0.15			**0.04**			0.30			**0.04**
Yes	1 (2.08%)	28 (8.64%)		1 (0.62%)	6 (5.04%)		7 (5.43%)	22 (9.05%)		1 (0.62%)	6 (5.04%)	
No	47 (97.92%)	296 (91.36%)		160 (99.38%)	113 (94.96%)		122 (94.57%)	221 (90.95%)		160 (99.38%)	113 (94.96%)	

Variable	Antibiotic stewardship						EBS pathway		
	Complicated AA			Non-complicated AA			Non-complicated AA		
	Yes *N* = 105	No *N* = 196	*p*-Value	Yes *N* = 106	No *N* = 174	*p*-Value	Yes *N* = 43	No *N* = 238	*p*-Value
Academic hospital			0.46			0.87			**0.02**
Yes	39 (37.14%)	63 (32.14%)		39 (36.79%)	61 (35.06%)		23 (53.49%)	78 (32.77%)	
No	66 (62.86%)	133 (67.86%)		67 (63.21%)	113 (64.94%)		20 (46.51%)	160 (67.23%)	
Dedicated EGS service			0.28			> 0.999			**0.01**
Yes	32 (30.48%)	47 (23.98%)		32 (30.19%)	53 (30.46%)		21 (48.84%)	64 (26.89%)	
No	73 (69.52%)	149 (76.02%)		74 (69.81%)	121 (69.54%)		22 (51.16%)	174 (73.11%)	
Age	41.76 (± 16.95)	42.45 (± 16.77)	0.69	30.96 (± 13.44)	35.33 (± 15.73)	**0.03**	32.84 (± 12.39)	33.82 (± 15.45)	0.85
BMI	24.26 (± 4.06)	25.31 (± 4.35)	0.05	23.79 (± 3.9)	24.07 (± 3.26)	0.15	23.98 (± 3.53)	23.98 (± 3.52)	0.96
ASA			0.35			0.82			0.36
1	55 (52.38%)	91 (46.43%)		69 (65.09%)	118 (67.82%)		30 (69.77%)	158 (66.39%)	
2	45 (42.86%)	87 (44.39%)		32 (30.19%)	50 (28.74%)		13 (30.23%)	69 (28.99%)	
3	4 (3.81%)	17 (8.67%)		5 (4.72%)	6 (3.45%)		0 (0.0%)	11 (4.62%)	
4	1 (0.95%)	1 (0.51%)							
Charlson comorbidity index	0.657 (± 1.31)	0.745 (± 1.21)	0.20	0.236 (± 0.698)	0.46 (± 1.24)	0.10	0.14 (± 0.413)	0.416 (± 1.15)	0.19
Alvarado score	7.1 (± 1.55)	6.89 (± 1.56)	0.37	5.79 (± 1.78)	6.14 (± 1.62)	0.08	6.42 (± 1.48)	5.94 (± 1.71)	0.11
AIR score	6.66 (± 1.76)	6.61 (± 1.93)	0.98	4.91 (± 1.8)	5.3 (± 1.74)	**0.03**	5.23 (± 1.81)	5.14 (± 1.77)	0.96
Operative time (minutes)	73.15 (± 28.17)	79.42 (± 30.07)	0.05	55.17 (± 16.88)	63.39 (± 23.05)	** < 0.001**	53.67 (± 17.92)	61.47 (± 21.64)	**0.02**
Difficulty grade			0.38			**0.00**			**0.03**
1	13 (12.38%)	20 (10.2%)		48 (45.28%)	45 (25.86%)		23 (53.49%)	70 (29.54%)	
2	34 (32.38%)	51 (26.02%)		37 (34.91%)	93 (53.45%)		17 (39.53%)	113 (47.68%)	
3	35 (33.33%)	60 (30.61%)		19 (17.92%)	30 (17.24%)		3 (6.98%)	46 (19.41%)	
4	18 (17.14%)	54 (27.55%)		1 (0.94%)	5 (2.87%)		0 (0.0%)	6 (2.53%)	
5	5 (4.76%)	10 (5.1%)		1 (0.94%)	1 (0.57%)		0 (0.0%)	2 (0.84%)	
Length of stay (days)	4.34 (± 1.34)	5.38 (± 2.7)	**0.01**	3.12 (± 1.14)	3.72 (± 1.53)	** < 0.001**	2.79 (± 1.06)	3.62 (± 1.45)	** < 0.001**
Clavien dindo classification of surgical complications > 2			0.43			> 0.999			> 0.999
Yes	1 (0.95%)	6 (3.06%)		0 (0.0%)	1 (0.57%)		0 (0.00%)	1 (0.42%)	
No	104 (99.05%)	190 (96.94%)		106 (100.0%)	173 (99.43%)		43 (100%)	237 (99.58%)	
Comprehensive complications Index	5.82 (± 8.68)	7.26 (± 13.28)	0.61	3.15 (± 4.48)	3.69 (± 7.23)	0.34	3.93 (± 5.03)	3.39 (± 6.53)	0.11
Health-care costs	3894.3 (± 948.39)	3967.27 (± 897.42)	0.56	2528.33 (± 279.57)	2677.66 (± 415.77)	** < 0.001**	2517.19 (± 375.95)	2639.65 (± 373.73)	**0.06**
Unplanned readmission			0.49			0.19			**0.01**
Yes	2 (2.33%)	7 (4.86%)		4 (4.71%)	2 (1.35%)		4 (10.26%)	2 (1.03%)	
No	84 (97.67%)	137 (95.14%)		81 (95.29%)	146 (98.65%)		35 (89.74%)	192 (98.97%)	
SSI overall			0.93			0.71			0.29
Yes	14 (13.33%)	24 (12.24%)		2 (1.89%)	5 (2.87%)		2 (4.65%)	5 (2.1%)	
No	91 (86.67%)	172 (87.76%)		104 (98.11%)	169 (97.13%)		41 (95.35%)	233 (97.9%)	
OS overall			> 0.999			0.71			
Yes	10 (9.52%)	19 (9.69%)		2 (1.89%)	5 (2.87%)		2 (4.65%)	5 (2.1%)	
No	95 (90.48%)	177 (90.31%)		104 (98.11%)	169 (97.13%)		41 (95.35%)	233 (97.9%)	

*EAES* European Association of Endoscopic Surgery, *POCUS* point of care ultrasound, *AIR* appendicitis inflammatory response, *AAS* adult appendicitis score, *QOE* quality of evidence

### Logistic regression analysis for predictors of higher compliance with items 4.8–12, 4.12–12, 7. 1–2

Hospitals with dedicated EGS units had a significantly higher chance of performing suction alone (*OR* = 2.79, [1.37; 5.68], *p* = 0.0046). Complicated AA (*OR* = 0.22, [0.14; 0.36], *p* < 0.0001), challenging LA with the need for external help (*OR* = 0.23, [0.08; 0.69], *p* = 0.0083), and the AIR score (*OR* = 0.85, [0.76; 0.96], *p* = 0.0073) were independent predictors of lower compliance.

The odds of following CPG for drain placement were higher in dedicated EGS service (*OR* = 2.07, [1.04; 4.12], *p* = 0.0374) and in academic hospitals (*OR* = 3.89, [2.04; 7.43], *p* 0.0001). Technically challenging LA with the need of external help *OR* = 0.14, [0.06; 0.32], *p* 0.0001), the need of a preoperative CT Scan (*OR* = 0.51, [0.32; 0.81], *p* = 0.0046) and the presence of a Complicated Appendicitis (*OR* = 0.61, [0.4; 0.92], *p* = 0.0181) were identified as independent barriers to evidence-based surgery for drain placement.

The last logistic regression model showed that longer procedures (*OR* = 0.26, [0.14; 0.46], *p* < 0.0001) negatively affected antibiotic stewardship in patients with uncomplicated AA, leading to an inappropriate prescription of postoperative antibiotics.

The analysis is reported in Table 3.

**Table 3:** Logistic regression model results for Items 4.8–12 on Peritoneal Irrigation (a), 4.12–12 on Abdominal Drains (b) and 7. 1–2 on postoperative antibiotics in uncomplicated AA (c)

Variable	Odds ratio [Confidence Intervals]	*p*-value
(a) LRM results to identify favouring factors and barriers to following recommendations on the use of PI		
Intercept	0.561 [0.107;2.96]	0.496
University hospital	0.993 [0.499;1.98]	0.984
**Dedicated EGS service**	**2.79 [1.37;5.68]**	**0.004**
Age	1.01 [0.986;1.03]	0.447
BMI	1.02 [0.951;1.09]	0.585
ASA	0.886 [0.251;3.13]	0.851
Charlson Comorbidity Index	0.685 [0.455;1.03]	0.068
**AIR score**	**0.851 [0.757;0.958]**	**0.007**
CT Scan	1.16 [0.686;1.97]	0.578
Operator: Resident	1.54 [0.981;2.42]	0.060
**Difficulty grade 4–5**	**0.23 [0.0772;0.685]**	**0.008**
**Complicated Appendicitis**	**0.221 [0.138;0.355]**	** < 0.001**
(b) LRM results to identify favouring factors and barriers to following recommendations on the use of AD		
Intercept	4.57 [1.75;11.94]	** < 0.001**
**University hospital**	**3.89 [2.04;7.43]**	** < 0.001**
**Dedicated EGS service**	**2.07 [1.04;4.12]**	** < 0.001**
Age	0.996 [0.981;1.01]	0.643
ASA	0.816 [0.307;2.17]	0.683
AIR score	0.922 [0.829;1.03]	0.133
**CT Scan**	**0.511 [0.321;0.814]**	**0.004**
**Time to surgery (hour)**	**1.02 [1.01;1.04]**	**0.006**
**Operative time (minutes)**	**0.979 [0.97;0.989]**	** < 0.001**
**Difficulty grade 4–5**	**0.139 [0.0599;0.324]**	** < 0.001**
**Complicated Appendicitis**	**0.605 [0.399;0.918]**	**0.018**
(c) LRM results to identify favouring factors and barriers to following recommendations on the correct use of postoperative antibiotics in uncomplicated AA		
Intercept	18.27 [4.12;81.02]	< 0.001
University Hospital	1.85 [0.8;4.3]	0.150
Dedicated ESS	0.511 [0.214;1.22]	0.130
Age	0.982 [0.961;1.0]	0.116
Alvarado Score	0.884 [0.753;1.04]	0.133
CT Scan	0.675 [0.327;1.39]	0.287
**Operative time (minutes)**	**0.981 [0.965;0.997]**	**0.017**
**Peritoneal Irrigation**	**0.257 [0.145;0.457]**	** < 0.001**
Drainage	0.747 [0.397;1.4]	0.364

*LRM* logistic regression model; *PI* peritoneal irrigation; *AD* abdominal drainage; *BMI* body mass index; *ASA* American Society of Anaesthesiologists, *CT* computed tomography, *ESS* emergency surgery service; *AA* acute appendicitis, *BMI* body mass index;

Significant associations are highlighted in bold

## Discussion

This multi-institutional audit explored the barriers and factors related to compliance with the most recent and cited CPG on AA, and the effects of poor adherence.

Our results on 653 adult patients undergoing LA for AA from 24 hospitals in northern Italy disclosed low adherence to the application of point-of-care ultrasound as the first approach, to recommendations on PI, drainage placement, and inappropriate postoperative AMT in uncomplicated and complicated AA. We showed the positive effect of teaching hospitals and dedicated EGS and the detrimental effect of surgeons’ perceptions during challenging LA. On the other hand, adherence was good for optimal in selection for cross-sectional imaging in young high-risk patients and in timing of surgery, considering the 24 h threshold. We reported that poor compliance may have a negative effect on surgical site infections for selected items. Low adherence led to longer hospitalizations with higher health-related costs.

Non-compliance in patients with uncomplicated disease is a red flag who are expected to have shorter hospitalizations, better postoperative outcomes and a lower impact on the health-care system [18].

In the first instance, we explored different outcomes considering an evidence-based framework in uncomplicated AA, with full compliance with timing for surgery within 24 h, avoidance of peritoneal irrigation and abdominal drainage and non-administration of postoperative AMT. Only 15.3% of patients with uncomplicated disease were treated following this evidence-based bundle, who benefitted from shorter surgeries, shorter hospitalizations and subsequent lower related costs. A recent review on the New England Journal of Medicine reported that patients with uncomplicated disease have a mean length of stay of 1.3 days [18] which is overall shorter than patients treated non-operatively as shown in recent meta-analysis of multiple randomized control trials and in the CODA trial published in 2020 on the New England Journal of Medicine [19, 20].

In our series patients undergoing appendectomy for the uncomplicated disease had a mean length of stay of 2.79 ± 1.06 days, with full compliance with the EBS framework, compared to 3.62 ± 1.06 days in case of non-compliance, with significantly higher related costs 2517 ± 375.95 euros versus 2639.65 ± 373.73.

Surprisingly, we report a non-negligible percentage of patients with uncomplicated AA, in which PI was used (57.5%) or a drain placed (35.7%). In both cases the univariate analysis disclosed higher rates of Organ Space SSI, but the low number of events may disclose a lack of clinical significance, similar evidence were reported by Bass et al. [9].

In the current surgical era in which the approach to uncomplicated disease is still a matter of strong debate, there should be no compromises in the quality of surgical management of these patients [21].

Albeit not following the EBS framework may not have a real clinical impact, other outcomes such as the length of the hospitalization and related costs should not be overlooked.

The impact on such benchmarks is multifactorial and is related to noncompliance with all the analysed items considered individually, regardless of appendicitis severity, or considered together in the EBS framework for uncomplicated diseases.

The financial burden of EGS was explored by Wohlgemuth and colleagues in the UK and by Ogola et al. in the US [1, 22]. Both studies disclosed that EGS constitutes a non-negligible portion of health-care-related costs. The projected costs of EGS are expected to increase by 20 to 45% in the future decades. One of the main drivers of costs increase is the growing age of the EGS population with a higher portion of frail patients. Identifying modifiable factors impacting measurable costs is critical, especially if clear indications from guidelines are available to improve patients’ hospitalizations and reduce the burden of care.

In uncomplicated and complicated AA, one of the main contributors to longer hospitalizations and higher costs can be the lack of antibiotic stewardship, influencing clinical outcomes in the short term [23], and increasing the public health burden of antimicrobial resistance (AMR).

Bass et al. showed that postoperative AMT was administered in 49 to 71.4% of patients with uncomplicated AA [9]. We confirmed this trend in 65% of patients. Unappropriated postoperative AMT is the main cause of AMR. In 2017, the first institutional task force against AMR was created following an audit by the European Center for Disease Control, which disclosed a hyper-endemic level of AMR. The report highlighted the lack of awareness among stakeholders who were not willing to take charge of the problem. Physicians declared to be poorly coordinated, not supported by institutions at any level, and not adequately led by chiefs in the implementation of local practices to improve the awareness of AMR [24–26].

In complicated AA, as shown in the ESTES snapshot [9], we confirmed low compliance (34.1%) to the 5-day postoperative AMT limit, with a mean duration of 6.77 days. A recent RCT published in Lancet reported the non-inferiority of a postoperative 2-day regimen compared to a 5-day regimen. Considering the overwhelming impact of AMR on in-hospital morbidity and mortality, these findings require further in-depth reflection on the knowledge-to-action gap in acute appendicitis [27].

We then performed an in-depth analysis of the decision-making on intra- and postoperative items that could affect patients’ outcomes and impact on related costs. Starting from a compliance assessment, we explored the risk factors for compliance and non-compliance to the recommendations on peritoneal irrigation, abdominal drains and postoperative antibiotics, the latter in uncomplicated appendicitis.

We report poor compliance to indications on PI and AD. More recent evidence confirmed that these practices did not prevent postoperative abscesses, increasing operative times, and were associated with higher re-operation rates [28–31]. We confirmed no advantages in complicated AA but possibly higher rates of OS SSI in uncomplicated AA with non-compliance. Furthermore, we disclosed higher expenditures related to non-compliance, regardless of the development of postoperative complications.

Challenging AA had a negative impact on compliance on PI and AD. We recently explored the reasons pushing surgeons toward PI, reporting how it was affected by the intraoperative overestimation of AA severity, which may certainly be negatively affected by surgical stressors [15].

A challenging LA independently leads to lower compliance. Surgeons’ reactions to “hostile” and outside-the-comfort zone AA can affect intraoperative choices. Chrouser and al. showed that intraoperative stressors can impair cognitive non-technical skills, affecting working memory capacity and decision-making [32].

The attitude towards evidence-based practices is higher for PI and AD in dedicated EGS units.

This evidence is supported by previous studies that demonstrated the positive impact of dedicated Acute Care Surgery (ACS) models on patient outcomes and healthcare costs [33–35].

Unfortunately, in Italy and other European countries, there is a lack of institutional emergency general surgery networks not allowing the implementation of benchmarking-based quality improvement programs [36].

The adherence to evidence-based recommendations on abdominal drainages was higher in teaching hospitals also. Our results on the impact of teaching hospitals can be explained considering that teaching hospitals may be more committed to trainees’ education on EBS. Considering emergency general surgery, a multicenter US snapshot showed very small absolute differences between teaching and non-teaching hospitals [37]. Better outcomes for emergency cases in non-teaching hospitals have been previously reported due to trainees' low experience and internal fragmentation of care before senior surgeons take the lead [38]. Finally, recent reports dispelled any doubt showing no difference in outcomes [39]. This evidence should encourage efforts toward EBS education for young trainees.

Furthermore, we analysed the process leading to the inadequate prescription of antibiotics after surgery for uncomplicated disease. We highlighted the negative impact of lengthy procedures and of the use of PI that can be associated with procedure perceived as more complex, and possibly with the occurrence of intraoperative stressors. Madani et al. created a framework to measure performance by considering the principles that influence surgical behaviours, showing that an emergency surgical procedure was perceived by a surgeon to be as stressful as the persistent occurrence of an intraoperative adverse event during an elective procedure [40].

The research field of knowledge transfer in clinical practice is complex and multifaceted.

Morris and colleagues in 2011 reported that the average time for research evidence to be implemented in daily clinical practice is 17 years [41].

Recent report exploring the current KTA gap disclosed that up to 50% of elective surgical patients are not treated following evidence-based practices, mostly due to a lack of awareness, this percentage may be higher in emergency surgical patients [42].

Narrowing this gap is one of the challenges in EBS. Diverse frameworks have been developed to analyse the process of knowledge implementation, identifying a critical phase in which local stakeholders must understand the context in which the new practice will be introduced and find new approaches along with potential barriers to implementation [43–47].

Our results, along with other recently published records, offer insights into real-world compliance with CPG and could be helpful in understanding the barriers to the application of EBS in the complex environment of ACS.

Non-profit projects are already available to speed up the KTA transition such as the *Hacking the Knowledge Gap Series* by the Canadian Institute of Gender and Health (IGH). Three major factors limiting the Knowledge Translation and Exchange (KTE) were identified: absence of time and skill; poor inclusion in dedicated updates, and the absence of grants and funding’s. The IGH fostered a collaboration between healthcare providers and professionals from marketing, communication and design areas, aiming to share knowledge and empower clinicians with new skills. Examples of possible solutions are public and clinical awareness campaigns, research-informed guidelines and e-health apps [48].

We identified three categories of barriers to knowledge implementation of EBS practices for AA with possible targets of interventions (Table 4).

**Table 4:** Modifiable and non-modifiable targets of intervention to narrow the knowledge-to-action gap

Category	Feature	Target of future interventions
Patients related factor	Non modifiable factor	Early and appropriate diagnosis Timely surgical approach
Environmental factor	Modifiable factor	Complex patients centralization in emergency general surgery service
		Hub and spoke networks for emergency general surgery
		Introduction of structured emergency general surgery verification programs
		Creation of institutional program for guidelines implementation with dedicated clinical bundles for knowledge translation from CPG and daily clinical practice critical appraisal
		Dedicated national and local dynamic programs to identify barriers to knowledge implementation
Surgeon-related factor	Modifiable and non-modifiable factor	Surgeons psychological empowerment to improve the ability to deal with adverse event and standardize the mental approach during emergency cases
		Encourage periodical update sessions on evidence-based practices
		Dedicated educational intervention on evidence-based medicine to improve the ability to understand CPG recommendations (basic statistics, methodology, consensus and guidelines building process)

We categorized barriers to evidence-based surgery and reported possible approaches to implement the knowledge-to-action transition

## Limitations

The nature and design of the study have some limitations. This was the first Italian multicenter trainee-led trial, and almost all residents were novices in clinical research. This approach aims to expose trainees to correct practices of patient enrolment, data collection and clinical monitoring of evidence-based practices.

Thus, we dedicate time to online and in-person tutoring to overcome the lack of experience despite this, we report a 20% rate of patients lost at follow up. The 6 months to 1 year clinical rotations led to multiple residents following a single patient and to the potential fragmentation of data collection and patients follow up.

One of the strongest limitations came from the Covid-19 outbreak. The pandemic has surely impacted the enrolment and follow up. Almost all the centers were at the core of the outbreak that hit Lombardy from 2020 to 2021. In some centers, surgeons and residents supported the local response working in respiratory units, taking time from surgery and surgical research. In other centers, residents were not allowed to work during the most intense phases of the outbreaks, making data collection almost impossible for entire months. We believe that, despite the harsh times, remarkable efforts were made to keep data collection activity.

Another limiting factor was the change of clinical behaviours during the pandemic. A European snapshot survey explored the evolution of the decision-making on AA during the early phases of the outbreak. There was a four-fold rise in non-operative approaches for uncomplicated and two-fold for complicated AA during the pandemic. More than one third of 709 surgeons declared a shift toward open approaches. The survey reported a global reduction of patients admitted with an increasing severity among those treated. These factors can explain the slow recruitment, considering our inclusion criteria, and the KTA gap [49].

Furthermore, there is a setting-related limitation that can also be a strength point. Compared to others, this study included also medium to small community hospitals serving peripheral areas. These centers probably reflect most real-world practices and are valuable targets for future interventions.

## Conclusions

In this study, we showed that the compliance to CPG on acute appendicitis is moderate to low. External and internal stressors can bias surgical decision-making, limiting the adherence to evidence-based practice with a real impact on hospitalizations and related costs. Moreover, a worrisome attitude toward antibiotic stewardship was disclosed. We identified barriers to KTE coming from the environment, the patient and the surgeon and proposed possible initiatives.

Future interventions will need to focus on: awareness of recent evidences and mental preparedness reducing intraoperative stress, tailored initiatives to foster a transversal emergency surgery culture, educational programs to narrow the gap among university and non-university centers and the creation of multi-institutional registries for benchmarking and define quality standards. Such efforts will play a pivotal role in knowledge implementation and improve patient outcomes. Future CPG will need to be integrated, considering aspects related to institutional and local barriers to the translation of evidence-based practices in surgery.

### Supplementary Information

Below is the link to the electronic supplementary material.

Supplementary file1 (DOCX 26 kb)
