# Supplementary material for: The burden of the knowledge-to-action gap in acute appendicitis
Source: Surg Endosc. 2023 Oct 26;37(12):9617–32. doi: 10.1007/s00464-023-10449-4 (PMC10709474; doi:10.1007/s00464-023-10449-4)
Supplement: Supplementary file 1 — Supplementary file1 (DOCX 26 kb) [file 464_2023_10449_MOESM1_ESM.docx]

| **Name** | **Surname** | **Email** | **ORCID** | **Affiliation** |
| --- | --- | --- | --- | --- |
| Giulia Arianna | Abruzzese | [Giuliarianna.abruzzese@unimi.it](mailto:Giuliarianna.abruzzese@unimi.it) |  | ASST GOM Niguarda, Milan, Italy |
| Francesca | Albanesi | [Francesca.albanesi@unimi.it](mailto:Francesca.albanesi@unimi.it) | [0000-0002-1898-1367](https://orcid.org/0000-0002-1898-1367) | ASST Fatebenefratelli-Sacco, Ospedale Fatebenefratelli, Milan, Italy |
| Michele | Altomare | [Michele.altomare@ospedaleniguarda.it](mailto:Michele.altomare@ospedaleniguarda.it) | 0000-0002-3879-2678 | ASST GOM Niguarda, Milan, Italy |
| Erika | Andreatta | [Erika.andreatta87@gmail.com](mailto:Erika.andreatta87@gmail.com) | [0000-0001-5130-6710](https://orcid.org/0000-0001-5130-6710) | ASST Santi Paolo e Carlo, Ospedale San Paolo, Milan, Italy |
| Ludovica | Baldari | [Ludovica.baldari@gmail.com](mailto:Ludovica.baldari@gmail.com) | [0000-0002-7029-8485](https://orcid.org/0000-0002-7029-8485) | Policlinico di Milano Ospedale Maggiore,  Fondazione IRCCS Ca' Granda, Milan, Italy |
| Laura | Benuzzi | [Laura.benuzzi@unimi.it](mailto:Laura.benuzzi@unimi.it) | [0000-0003-1542-2807](https://orcid.org/0000-0003-1542-2807) | ASST GOM Niguarda, Milan, Italy |
| Emanuele | Bevilaqua | [Emanuele.bevilaqua@unimi.it](mailto:Emanuele.bevilaqua@unimi.it) | [0000-0002-5998-8514](https://orcid.org/0000-0002-5998-8514) | ASST Nord Milano, Ospedale di Sesto San Giovanni,  Sesto San Giovanni Italy |
| Alessandro Michele | Bonomi | [Alessandro.bonomi@unimi.it](mailto:Alessandro.bonomi@unimi.it) | [0000-0002-7248-197X](https://orcid.org/0000-0002-7248-197X) | ASST Brianza, Ospedale di Vimercate, Vimercate, Italy |
| Greta | Brachetti | [Greta.brachetti@unimi.it](mailto:Greta.brachetti@unimi.it) |  | ASST Brianza, Ospedale di Vimercate, Vimercate, Italy |
| Giulia | Cannavale | [Giulia.cannavale@gmail.com](mailto:Giulia.cannavale@gmail.com) |  | IRCCS Multimedica, Sesto San Giovanni, Sesto San Giovanni, Italy |
| Andrea Piero | Chierici | [Andreapiero.chierici@gmail.com](mailto:Andreapiero.chierici@gmail.com) | [0000-0003-1475-4500](https://orcid.org/0000-0003-1475-4500) | ASST Brianza, Ospedale di Vimercate, Vimercate, Italy |
| Stefano PB | Cioffi | [Stefanopiero.cioffi@ospedaleniguarda.it](mailto:Stefanopiero.cioffi@ospedaleniguarda.it) | 0000-0002-5453-8901 | ASST GOM Niguarda, Milan, Italy |
| Riccardo | Cirelli | [Riccardo.cirelli@unimi.it](mailto:Riccardo.cirelli@unimi.it) | [0000-0003-0925-507X](https://orcid.org/0000-0003-0925-507X) | IRCCS Multimedica, Sesto San Giovanni, Sesto San Giovanni, Italy |
| Gaia | Colletti | [Gaia.colletti@unimi.it](mailto:Gaia.colletti@unimi.it) | [0000-0001-9859-836X](https://orcid.org/0000-0001-9859-836X) | Ospedale “San Leopoldo Mandic” di Merate, Merate, Italy |
| Vera | D'abrosca | [Vera.dabrosca@unimi.it](mailto:Vera.dabrosca@unimi.it) |  | Policlinico di Milano Ospedale Maggiore,  Fondazione IRCCS Ca' Granda, Milan, Italy |
| Piergiorgio | Danelli | [Piergiorgio.danelli@unimi.it](mailto:Piergiorgio.danelli@unimi.it) | [0000-0003-1461-9835](https://orcid.org/0000-0003-1461-9835) | ASST Fatebenefratelli-Sacco, Ospedale Fatebenefratelli, Milan, Italy |
| Luca | Del Prete | [Luca.delprete@policlinico.mi.it](mailto:Luca.delprete@policlinico.mi.it) | [0000-0003-0268-3266](https://orcid.org/0000-0003-0268-3266) | ASST Rhodense, Ospedale di Rho, Rho, Italy |
| Francesco | Di Capua | [Francesc.dicapua@gmail.com](mailto:Francesc.dicapua@gmail.com) | [0000-0001-6636-8172](https://orcid.org/0000-0001-6636-8172) | ASST Fatebenefratelli-Sacco, Ospedale Fatebenefratelli, Milan, Italy |
| Francesca | Di Vittorio | [Divittorio.francesca@hsr.it](mailto:Divittorio.francesca@hsr.it) | [0000-0002-8978-3798](https://orcid.org/0000-0002-8978-3798) | ASST GOM Niguarda, Milan, Italy |
| Davide | Ferrari | [Davide.ferrari@unimi.it](mailto:Davide.ferrari@unimi.it) |  | Policlinico San Marco di Zingonia, Osio Sotto, Italy |
| Luca | Ferrario | [Luca.ferrario1@unimi.it](mailto:Luca.ferrario1@unimi.it) | [0000-0002-3652-3255](https://orcid.org/0000-0002-3652-3255) | ASST GOM Niguarda, Milan, Italy |
| Laura | Fiore | Laurafiore@msn.com | [0000-0002-7360-3625](https://orcid.org/0000-0002-7360-3625) | ASST Santi Paolo e Carlo, Ospedale San Paolo, Milan, Italy |
| Colomba | Frattaruolo | [Colomba.frattaruolo@unimi.it](mailto:Colomba.frattaruolo@unimi.it) | [0000-0001-6165-2641](https://orcid.org/0000-0001-6165-2641) | ASST GOM Niguarda, Milan, Italy |
| Caterina | Froiio | [Caterina.froiio@unimi.it](mailto:Caterina.froiio@unimi.it) | [0000-0003-1018-3691](https://orcid.org/0000-0003-1018-3691) | ASST Santi Paolo e Carlo, Ospedale San Paolo, Milan, Italy |
| Ludovica | Gibelli | [Ludovica.gibelli@unimi.it](mailto:Ludovica.gibelli@unimi.it) | [0000-0002-1230-0057](https://orcid.org/0000-0002-1230-0057) | ASST GOM Niguarda, Milan, Italy |
| Irene | Giusti | [Irene.giusti@unimi.it](mailto:Irene.giusti@unimi.it) | [0000-0002-6930-3144](https://orcid.org/0000-0002-6930-3144) | ASST Santi Paolo e Carlo, Ospedale San Paolo, Milan, Italy |
| Ugo | Giustizieri | [Ugo.giustizieri@unimi.it](mailto:Ugo.giustizieri@unimi.it) | [0000-0003-3100-4496](https://orcid.org/0000-0003-3100-4496) | Ospedale “San Leopoldo Mandic” di Merate, Merate, Italy |
| Samuele | Grandi | [Samuele.grandi@unimi.it](mailto:Samuele.grandi@unimi.it) | [0000-0002-9257-0240](https://orcid.org/0000-0002-9257-0240) | ASST Fatebenefratelli-Sacco, Ospedale Sacco, Milan, Italy |
| Stefano | Granieri | [Steff.granieri@gmail.com](mailto:Steff.granieri@gmail.com) | [0000-0001-9167-3400](https://orcid.org/0000-0001-9167-3400) | ASST Brianza, Ospedale di Vimercate, Vimercate, Italy |
| Giulio | Iacob | [Giulio.iacob@unimi.it](mailto:Giulio.iacob@unimi.it) | [0000-0002-8929-1304](https://orcid.org/0000-0002-8929-1304) | Ospedale “Sacra Famiglia” Fatebenefratelli di Erba, Erba Italy |
| Alessia | Kersik | [Alessia.kersik@unimi.it](mailto:Alessia.kersik@unimi.it) | [0000-0002-2888-9572](https://orcid.org/0000-0002-2888-9572) | ASST Brianza, Ospedale di Vimercate, Vimercate, Italy |
| Pietro | Lombardi | [Pietro.lombardi@humanitas.it](mailto:Pietro.lombardi@humanitas.it) | [0000-0002-2633-7977](https://orcid.org/0000-0002-2633-7977) | ASST Rhodense, Ospedale di Garbagnate Milanese,  Garbagnate Milanese, Italy |
| Marco | Longhi | [Marco.longhi@asst-lodi.it](mailto:Marco.longhi@asst-lodi.it) | [0000-0001-9581-4450](https://orcid.org/0000-0001-9581-4450) | ASST Melegnano Martesana, Ospedale Vizzolo Predabissi, Italy |
| Leonardo | Lorusso | [Leonardo.lorusso@unimi.it](mailto:Leonardo.lorusso@unimi.it) | [0000-0001-9581-4450](https://orcid.org/0000-0001-9581-4450) | Ospedale Bolognini, Seriate, Italy |
| Michele | Manara | [michele.manara@unimi.it](mailto:michele.manara@unimi.it) |  | ASST Rhodense, Ospedale di Garbagnate Milanese,  Garbagnate Milanese, Italy |
| Elena | Manzo | Elena.manzo@unimi.it | [0000-0001-6558-5431](https://orcid.org/0000-0001-6558-5431) | Policlinico di Milano Ospedale Maggiore,  Fondazione IRCCS Ca' Granda, Milan, Italy |
| Jacopo Nicolo | Marin | [Jacopo.marin@unimi.it](mailto:Jacopo.marin@unimi.it) | [0000-0001-6951-6695](https://orcid.org/0000-0001-6951-6695) | ASST GOM Niguarda, Milan, Italy |
| Marianna | Maspero | [Marianna.maspero@unimi.it](mailto:Marianna.maspero@unimi.it) | [0000-0002-7589-4489](https://orcid.org/0000-0002-7589-4489) | ASST GOM Niguarda, Milan, Italy |
| Valentina | Messina | [Valentina.messima@unimi.it](mailto:Valentina.messima@unimi.it) | [0000-0003-0127-4161](https://orcid.org/0000-0003-0127-4161) | ASST GOM Niguarda, Milan, Italy |
| Pamela | Milito | [Pamela.milito@unimi.it](mailto:Pamela.milito@unimi.it) | [0000-0002-5926-3943](https://orcid.org/0000-0002-5926-3943) | IRCCS Policlinico San Donato, San Donato Milanese, Italy |
| Mattia | Molteni | [Molteni.mattia@hsr.it](mailto:Molteni.mattia@hsr.it) | [0000-0003-1471-439X](https://orcid.org/0000-0003-1471-439X) | ASST GOM Niguarda, Milan, Italy |
| Eleonora | Monti | [Eleonora.monti44@gmail.com](mailto:Eleonora.monti44@gmail.com) | [0000-0001-8178-7789](https://orcid.org/0000-0001-8178-7789) | Ospedale “Sacra Famiglia” Fatebenefratelli di Erba, Erba Italy |
| Vincenzo | Nicastro | [Vincenzo.nicastro@unimi.it](mailto:Vincenzo.nicastro@unimi.it) | [0000-0002-0893-9640](https://orcid.org/0000-0002-0893-9640) | ASST Santi Paolo e Carlo, Ospedale San Paolo, Milan, Italy |
| Giorgio | Novelli | [Giorgio.novelli@unimi.it](mailto:Giorgio.novelli@unimi.it) | [0000-0001-9506-0871](https://orcid.org/0000-0001-9506-0871) | ASST GOM Niguarda, Milan, Italy |
| Sissi | Paleino | [Sissi.paleino@asst-brianza.it](mailto:Sissi.paleino@asst-brianza.it) | [0000-0001-7315-763X](https://orcid.org/0000-0001-7315-763X) | ASST Brianza, Ospedale di Vimercate, Vimercate, Italy |
| Silvia | Pavesi | [Silvia.pavesi@unimi.it](mailto:Silvia.pavesi@unimi.it) |  | Policlinico di Milano Ospedale Maggiore,  Fondazione IRCCS Ca' Granda, Milan, Italy |
| Carolina | Perali | [Carolina.perali@unimi.it](mailto:Carolina.perali@unimi.it) |  | ASST GOM Niguarda, Milan, Italy |
| Isabella | Pezzoli | [Isabella.pezzoli@unimi.it](mailto:Isabella.pezzoli@unimi.it) | [0000-0003-0498-8258](https://orcid.org/0000-0003-0498-8258) | ASST Fatebenefratelli-Sacco, Ospedale Sacco, Milan, Italy |
| Roberta | Ragozzino | [Roberta.ragozzino@ospedaleniguarda.it](mailto:Roberta.ragozzino@ospedaleniguarda.it) | [0000-0002-0723-397X](https://orcid.org/0000-0002-0723-397X) | ASST GOM Niguarda, Milan, Italy |
| Giuliano | Santolamazza | [Giuliano.santolamazza@unimi.it](mailto:Giuliano.santolamazza@unimi.it) | [0000-0002-0656-4849](https://orcid.org/0000-0002-0656-4849) | ASST GOM Niguarda, Milan, Italy |
| Luca | Scaravilli | [Luca.scaravilli@unimi.it](mailto:Luca.scaravilli@unimi.it) | [0000-0002-1326-1862](https://orcid.org/0000-0002-1326-1862) | ASST GOM Niguarda, Milan, Italy |
| Andrea | Spota | [Andrea.spota@ospedaleniguarda.it](mailto:Andrea.spota@ospedaleniguarda.it) | [0000-0003-2288-361X](https://orcid.org/0000-0003-2288-361X) | ASST GOM Niguarda, Milan, Italy |
| Gilda | Tornatore | [Gilda.tornatore@gmail.com](mailto:Gilda.tornatore@gmail.com) | 0000-0001-7947-4572 | ASST Santi Paolo e Carlo, Ospedale San Paolo, Milan, Italy |
| Francesco | Toti | [Francesco.toti@unimi.it](mailto:Francesco.toti@unimi.it) | [0000-0001-9105-4431](https://orcid.org/0000-0001-9105-4431) | ASST Fatebenefratelli-Sacco, Ospedale Sacco, Milan, Italy |
| Vincenzo | Tripodi | [Vincenzo.tripodi@unimi.it](mailto:Vincenzo.tripodi@unimi.it) | [0000-0001-6611-6477](https://orcid.org/0000-0001-6611-6477) | Policlinico di Milano Ospedale Maggiore,  Fondazione IRCCS Ca' Granda, Milan, Italy |
| Elisa | Vaterlini | [Elisa.vaterlini@libero.it](mailto:Elisa.vaterlini@libero.it) |  | ASST Milanese Ovest,  Ospedale Giuseppe Fornaroli, Magenta, Italy |
| Barbara | Vignati | [Barbara.vignati@unimi.it](mailto:Barbara.vignati@unimi.it) | [0000-0003-0454-2432](https://orcid.org/0000-0003-0454-2432) | Ospedale Valduce, Como, Italy |
| Cecilia | Maina | [maina.cecilia@hsr.it](mailto:maina.cecilia@hsr.it) | [0000-0002-8754-1007](https://orcid.org/0000-0002-8754-1007) | ASST GOM Niguarda, Milan, Italy |
| Alessandra | Borghi | [alessandra.borghi@unimi.it](mailto:alessandra.borghi@unimi.it) | 0000-0001-8827-4038 | ASST GOM Niguarda, Milan, Italy |
| Marco | Realis Luc | [marco.realis@unimi.it](mailto:marco.realis@unimi.it) |  | ASST GOM Niguarda, Milan, Italy |
| Paolo | Pizzini | [paolo.pizzini@unimi.it](mailto:paolo.pizzini@unimi.it) | 0000-0001-9860-9356 | ASST GOM Niguarda, Milan, Italy |
| Riccardo | Masserano | [riccardo.masserano@unimi.it](mailto:riccardo.masserano@unimi.it) |  | ASST GOM Niguarda, Milan, Italy |
| Marta | Maistri | [marta.maistri@hotmail.com](mailto:marta.maistri@hotmail.com) |  | ASST GOM Niguarda, Milan, Italy |
| Laura | Traballi | [lauritrbll@gmail.com](mailto:lauritrbll@gmail.com) |  | ASST GOM Niguarda, Milan, Italy |
| Francesco | Cammarata | [francesco.cammarata1@unimi.it](mailto:francesco.cammarata1@unimi.it) | [0000-0002-4055-8485](https://orcid.org/0000-0002-4055-8485) | ASST GOM Niguarda, Milan, Italy |
| Alvino | Boero | [alvino.boero@unimi.it](mailto:alvino.boero@unimi.it) |  | ASST GOM Niguarda, Milan, Italy |
| Davide | Socci | [socci.davide@hsr.it](mailto:socci.davide@hsr.it) | [0000-0001-6047-7298](https://orcid.org/0000-0001-6047-7298) | ASST GOM Niguarda, Milan, Italy |
| Beatrice | Zamburlini | [bea.zamburlini@gmail.com](mailto:bea.zamburlini@gmail.com) | [0000-0002-8015-0468](https://orcid.org/0000-0002-8015-0468) | ASST GOM Niguarda, Milan, Italy |
| Margherita | Carbonaro | [margherita.carbonaro@unimi.it](mailto:margherita.carbonaro@unimi.it) | [0000-0001-5134-2797](https://orcid.org/0000-0001-5134-2797) | ASST GOM Niguarda, Milan, Italy |
| Martina | Pellegrini | [martina.pellegrini@unimi.it](mailto:martina.pellegrini@unimi.it) |  | ASST GOM Niguarda, Milan, Italy |
